# Online keyword searching in three countries and languages reflects different perceptions and behaviors in response to the name of the novel coronavirus disease

**DOI:** 10.7189/jogh.10.020339

**Published:** 2020-12

**Authors:** Renyu Liu, Jonathan R Gavrin, Lee A Fleisher

**Affiliations:** 1 Department of Anesthesiology and Critical Care, Perelman School of Medicine at the University of Pennsylvania, Philadelphia, Pennsylvania, USA

In this viewpoint, we used three online search engines in three different languages: Google used in the United States and Japan, and Baidu used predominantly in China to investigate the perceptions and behaviors in response to the name of the novel coronavirus disease (COVID-19) in different countries and cultures. All engines offer statistical data on the trends of online searches: Google Trends in English and Japanese, and Baidu Index in Chinese. We found that the medical term “COVID-19” is not commonly used as a search keyword in any of the three countries we examined. There is a clear disconnect between “COVID-19” with either “pneumonia” (the severity of the disease and the effect on the respiratory system) or “mask” (the tool to defend against the pandemic). “Coronavirus” is the most used keyword in the US and is associated with “stock” rather than “mask”. “Coronavirus” is the most used keyword in Japan and is associated with “mask” rather than “stock”. “Pneumonia” is used China and is associated with “mask” rather than “stock”. Such differences reflect different perceptions of the disease and could potentially contribute to the different dynamics of public behavior influencing viral spread and containment.

**Figure:**
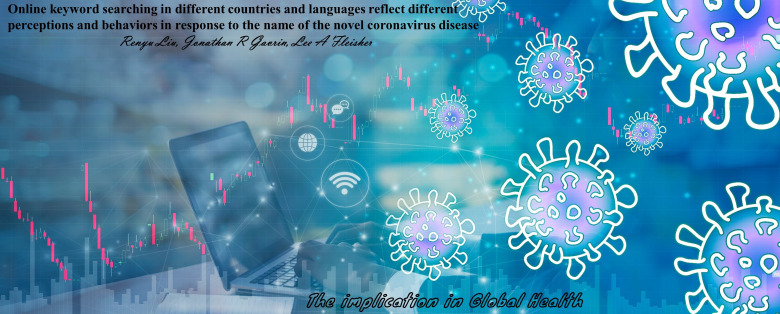
Photo: We use this art work to represent our work to demonstrate that online keyword searching trends reflect perceptions and behaviors in response to the name of the novel coronavirus disease. The original image is obtained by Dr Renyu Liu from Shutterstock (https://www.shutterstock.com/home) with an Enhanced License for unlimited editing and usage).

SARS-CoV-2 induced disease (COVID-19) is the medical name used to indicate that this is a disease caused by the novel coronavirus that occurred in 2019 [1,2]. Public perception and its resultant actions may help or hamper efforts to contain the spread of the disease or the virus. We hypothesized that different countries, using different languages, might use slightly different terminologies related to COVID-19 to search information online that may have profound impacts on disease spread or containment. Searching patterns have been used to predict and monitor the progression of an outbreak such as the current COVID-19 [3]. We utilized the unique search engines in different countries and in different languages to investigate the online behavior of keywords as they relate to COVID-19: Google in the United States, Google Japan in Japan, and Baidu in China. All the engines offer statistical data on the trends of online searches: Google Trends (https://trends.google.com/trends/) in the USA, Google Trends Japan (https://trends.google.co.jp/trends/?geo=JP) in Japan, and Baidu Index (http://index.baidu.com/v2/index.html#/) in China. We obtained data points from a 90-day period (daily data or weekly average from Jan 15, 2020 to April 15, 2020 automatically reported by the engine). The terms in three different languages used for searching are “coronavirus” (the cause of the disease), “pneumonia” (the severity of the disease and the connection with the respiratory system), “mask” (the key tool to defend against the pandemic), “COVID-19” (the official name of the disease), “stock” (the term related to the economy), “flu” (potential confusion associated with the coronavirus disease). The data collection spanned April 14 to April 16, 2020. To keep the consistency among three languages, we used “stock” as the key words in English. The correlation between the volume of searches was analyzed using linear regression with Graphpad (version 8.2.1, San Diego, CA). *P* less than 0.05 was considered statistically significant for correlation.

**Figure 1:**
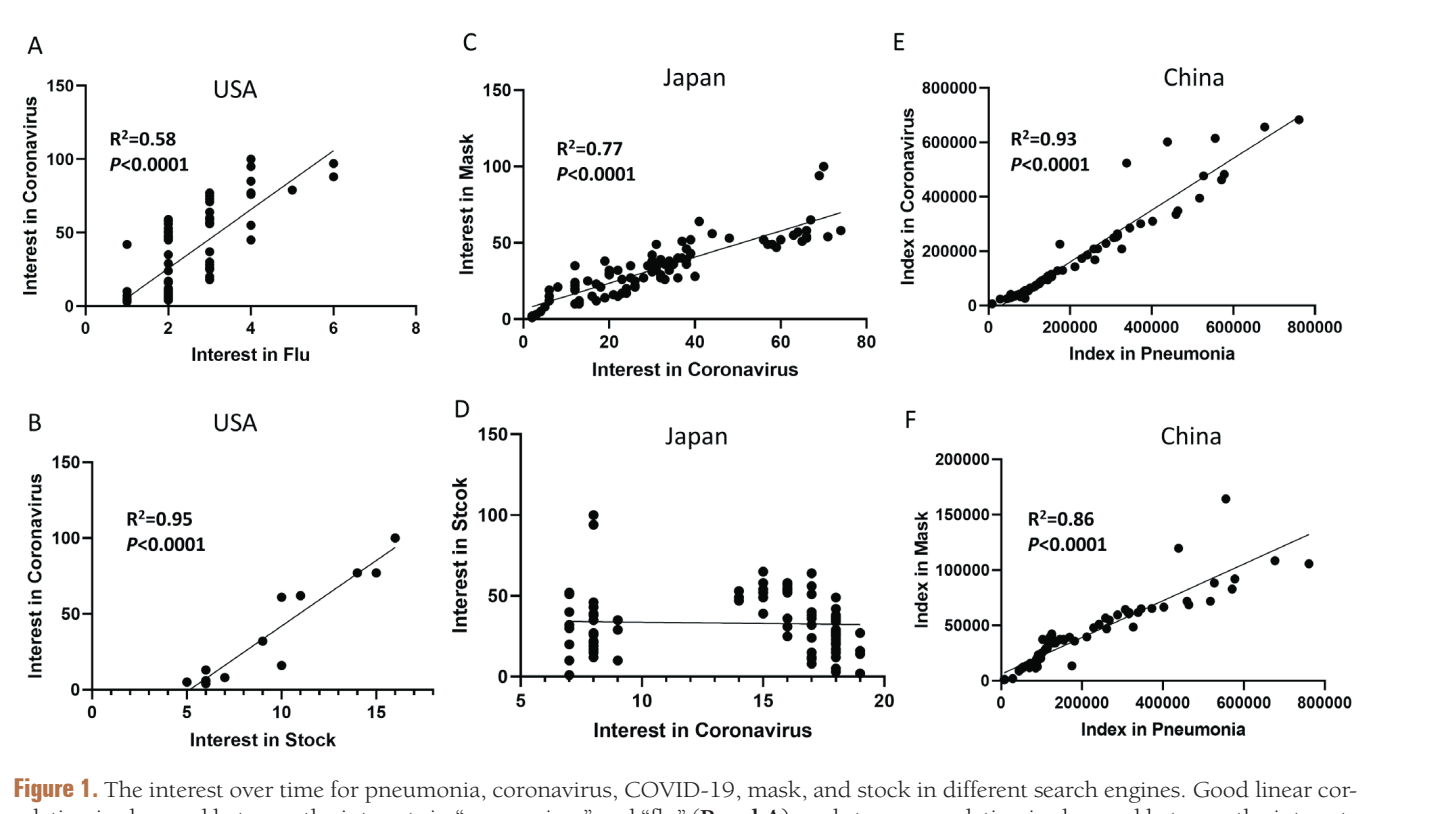
The interest over time for pneumonia, coronavirus, COVID-19, mask, and stock in different search engines. Good linear correlation is observed between the interests in “coronavirus” and “flu” (Panel A), and strong correlation is observed between the interests on “coronavirus” and “stock” during the outbreak (Panel B) in the US. Note that the weekly average data are used, and the difference in the scale is due to changes in the relative search volumes. Larger numbers in the trend scale indicate relatively larger volumes of searching. In Japan, strong linear correlation is observed between the interest in “coronavirus” and interest in “mask” (Panel C), and no correlation is observed between “coronavirus” and “stock” during the outbreak (**Panel D**). In China, there is clear pattern similarity between “coronavirus” and “pneumonia”. Strong linear correlation is observed between the searching Index for “coronavirus” and that for “pneumonia” (**Panel E**), and between the searching indices for “pneumonia” and for “mask” (**Panel F**).

In the US, the most searched keyword in Google was “coronavirus”. Searches for “pneumonia” and “COVID-19” remained flat without significant spikes. There is a clear disconnect between “pneumonia” and “coronavirus”. The search on “coronavirus” peaked between March 12 and March 16, the week prior to the initial stay at home orders in the United States. The search volume for “coronavirus” correlated well with that for “flu” (**Figure 1**, Panel A). Searches on “stock” fluctuated along with that for “coronavirus”; there is correlation between the searching of “coronavirus” and searching “stock” (**Figure 1**, Panel B). The trend in searching “stock” correlates well with that for “stock market”. The search of “coronavirus” pointed to some pages discussing “flu”. Searches of “mask” increased more recently, and peaked on April 4, 2020, the day after the Centers for Disease Control and Prevention recommended wearing masks. There is no correlation between searches for “coronavirus” and searches for “mask”.

In Japan, the most searched keyword in Google was “coronavirus”. Searches for “COVID-19” remained very low and flat without significant spikes as compared to that for “coronavirus”. There is a spike in the search for pneumonia in the early phase of the outbreak and it correlated linearly with that of “coronavirus” before the name “COVID-19” was announced (R^2^ = 0.88, *P* < 0.0001). The correlation disappeared after the official name was announced. The search on “coronavirus” peaked April 4, 2020. The search volume for “coronavirus” correlated well with that for “mask” (**Figure 1**, Panel C). There is no correlation between the searches of “coronavirus” and “stock” (**Figure 1**, Panel D). To determine if mask culture is the key driving factor of the “mask” search in Japan, we compared the trends in searches of “flu” and “mask”. There is a clear spike in the trends of searching for “flu” from 2004 on an annual basis; the spike in the H1N1 flu outbreak is shown. However, the dramatic surge in the search of “mask” appeared only recently during the coronavirus outbreak.

In China, the most searched keyword related to the coronavirus was “pneumonia”. The search on “pneumonia” started to increase from January 19, 2020, and peaked on January 25, 2020, just after the shut-down of Wuhan, China. The search on “coronavirus” peaked at a similar time as that on “pneumonia”. There is strong correlation between the searches for “coronavirus” and those for “pneumonia” (**Figure 1**, Panel E). The search on COVID-19 only occupied a small portion of the search volume compared to “pneumonia” and “coronavirus”. In China, the search on “masks” peaked at the same time as that for “pneumonia”; the frequency of searches for “pneumonia” correlates with searches of “mask” (**Figure 1**, Panel F). The search on “stock” remained flat during the epidemic time in China.

Using COVID-19 as the key word in Google, “COVID-19” is abbreviated as “coronavirus” in many places on the front page. “Coronavirus” appeared 31 times, “pneumonia” appeared only twice. Using “COVID-19” as the keyword in Baidu, “COVID-19” was translated to “Novel Coronavirus Pneumonia”, the original name used by Chinese medical experts and media.

This novel coronavirus-induced disease was initially named as novel coronavirus “pneumonia” in China. People in China continued using this term as demonstrated in the search volumes using Baidu in China, even after the World Health Organization (WHO) designated the official name of “coronavirus disease (COVID-19)” on 11 February 2020, along with a new name of the virus as “severe acute respiratory syndrome coronavirus 2 (SARS-CoV-2)” [4]. The WHO website stated that “From a risk communications perspective, using the name SARS can have unintended consequences in terms of creating unnecessary fear for some populations, especially in Asia which was worst affected by the SARS outbreak in 2003”. The attempt to avoid using the SARS term may accurately reflect the fact that COVID-19 not only affects the respiratory system, but also other vital systems including the neurological system [5]. However, the name of COVID-19 removed direct linkage of the disease with the respiratory syndrome and pneumonia. This may have influenced the pubic impression of the risk and profound threat of the disease.

Given the different searching patterns between Google in Japan and the USA, there is a clear disconnect between “coronavirus” and “pneumonia”, especially in the USA. The search volume of “COVID-19” is relatively low in both Google and Baidu. Misunderstanding or miscommunication related to the name of “COVID-19” was reported by CNN on April 15, 2020 [6]. Many people thought that it was just another common flu, was not associated with increased risk, and they are vigorously against physical distancing and mask wearing. In contrast, “pneumonia” continues to be used in China by the public and lay media. “COVID-19” is actually translated into “Novel Coronavirus Pneumonia”, the original name the Chinese were using. We believe that this most likely led to a better-informed public of the direct link between the virus and pneumonia. With respect to pneumonia, the direct response of the people in Asia is to wear a mask in order to blunt the possibility of transmitting or contracting the disease. Thus, it is not surprising that searches for “mask” on the web correlate well with searches for “pneumonia”. In Japan, the search trends of “coronavirus” matched well that of “mask”; there was a strong linear association between the search trends of “coronavirus” and “mask”, and between that of “coronavirus” and “pneumonia”. This could potentially be due to the unique mask wearing culture formed in Japan after the H1N1 influenza epidemic of 2009 [7]. However, the search trends of “mask” along with “flu” from 2004 in Japan do not support such an assumption. The search on “mask” only soared dramatically in the recent coronavirus outbreak. We hypothesize that the early surge in search of pneumonia might have played a critical role. Taken together, we think that the original name of “Novel Coronavirus Pneumonia” (NCP) was better understood by the public. The importance of naming a new disease and its influence on public behavior should be considered in the future.

While it is critical to think about economics early on during a pandemic, the first priority is to control the spread of disease. The increase in searches for “stock” and “stock market” occurred after 24 February 2020, when the Dow Jones Industrial Average and Financial Times Stock Exchange 100 Index dropped more than 3% as the coronavirus outbreak spread, worsening significantly outside China [8]. Therefore, our findings in the United States likely reflect public anxiety to coronavirus’ spread and the effect on the economy. The fact that “mask” was not a concern of the people in the English-speaking world, until later, may have had profound public health implications.

The medical term “COVID-19” is not commonly used as a search keyword by laypeople. “Coronavirus” is used in the US and is associated with “stock” rather than “mask”. “Coronavirus” is used in Japan, and is associated with “mask” rather than “stock”. “Pneumonia” is used China, which is associated with “mask” rather than “stock”. The dissimilarities likely reflect different perceptions of the disease and could potentially contribute to the different patterns of viral spread and containment among the countries. We postulate that different terminologies could potentially generate disparate psychological impacts and social behaviors, leading to different public health consequences.
